# Chinese SLE Treatment and Research Group Registry (CSTAR) XIII: prevalence and risk factors for chronic scarring alopecia in patients with systemic lupus erythematosus

**DOI:** 10.1186/s13075-020-02407-5

**Published:** 2021-01-11

**Authors:** Yirong Xiang, Mengtao Li, Hui Luo, Yanhong Wang, Xinwang Duan, Cheng Zhao, Feng Zhan, Zhenbiao Wu, Hongbin Li, Min Yang, Jian Xu, Wei Wei, Lijun Wu, Hanxiao You, Junyan Qian, Xiaoxi Yang, Can Huang, Jiuliang Zhao, Qian Wang, Xiaomei Leng, Xinping Tian, Yan Zhao, Xiaofeng Zeng

**Affiliations:** 1 Department of Rheumatology, State Key Laboratory of Complex Severe and Rare Diseases, National Clinical Research Center for Dermatologic and Immunologic Diseases (NCRC-DID), Key Laboratory of Rheumatology and Clinical Immunology, Peking Union Medical College Hospital, Chinese Academy of Medical Science and Peking Union Medical College, No. 1 Shuaifuyuan, Wangfujing Ave, Beijing, 100730 China; 2 Department of Rheumatology, Xiangya Hospital, Central South University, Changsha, China; 3 Department of Epidemiology and Bio-statistics, Institute of Basic Medical Sciences, China Academy of Medical Sciences & Peking Union Medical College, Beijing, China; 4 Department of Rheumatology, The Second Affiliated Hospital of Nanchang University, Nanchang, China; 5 Department of Rheumatology and Clinical Immunology, The First Affiliated Hospital of Guangxi Medical University, Nanning, China; 6 Department of Rheumatology, Hainan General Hospital, Haikou, China; 7 Department of Clinical Immunology and Rheumatology, Xijing Hospital Affiliated to the Fourth Military Medical University, Xi’an, China; 8 Department of Rheumatology, Affiliated Hospital of Inner Mongolia Medical College, Hohhot, China; 9 Department of Rheumatology and Immunology, Nanfang Hospital, Southern Medical University, Guangzhou, China; 10 Department of Rheumatology, First Affiliated Hospital of Kunming Medical University, Kunming, China; 11 Department of Rheumatology, Tianjin Medical University General Hospital, Tianjin, China; 12 Department of Rheumatology, People Hospital of Xinjiang Uygur Autonomous Region, Urumchi, China

**Keywords:** Scarring alopecia, Systemic lupus erythematosus, Risk factor

## Abstract

**Background:**

Scarring alopecia in systemic lupus erythematosus (SLE) patients caused reduced life quality and prolonged disease course. This case-control study aims to survey the prevalence of scarring alopecia during the disease course of SLE and evaluate the risk factors for scarring alopecia in Chinese SLE patients.

**Methods:**

SLE patients in Chinese SLE treatment and Research group (CSTAR) were recruited. Scarring alopecia was defined according to SLICC/ACR-DI which was collected during follow-up visits or via self-reported questionnaires. We collected demographic characteristics, common comorbidities, autoantibody profiles, disease activity status, major organ involvements, and treatment strategies of these patients at registry. Univariate and multivariate logistic regression analyses were used to investigate the risk factors for scarring alopecia.

**Results:**

We recruited 4792 SLE patients, and 374 (7.80%) patients had scarring alopecia. Mucocutaneous lesions (OR 2.062, *p* < 0.001), high SLICC/ACR-DI (OR 1.409, *p* < 0.001), and positive anti-Sm (OR 1.374, *p* = 0.029) were risk factors for scarring alopecia, while renal (OR 0.714, *p* = 0.028) and cardio-respiratory involvements (OR 0.347, *p* = 0.044), and immunosuppressant treatment (OR 0.675, *p* < 0.001) were significantly negative associated with it.

**Conclusions:**

The prevalence of scarring alopecia in SLE patients is 7.80%. Active treatment strategies should be adopted to prevent scarring alopecia occurring.

## Background

Systemic lupus erythematosus (SLE) is a chronic autoimmune disease characterized by high levels of autoantibody and multi-organ tissue damage [1]. Hair and scalp involvement is a common manifestation, presenting in more than half of the patients during the disease course [2–4]. Alopecia, its typical symptom, manifests with non-scarring or scarring. Non-scarring alopecia, more commonly, is defined in 2012 Systemic Lupus International Collaborating Clinics (SLICC) classification criteria for SLE as diffuse thinning or hair fragility with visible broken hairs [5, 6]. Regarding scarring alopecia, discoid lupus erythematosus (DLE) runs a chronic course and ends up with distinctive pattern of scarring hair loss, which is included as one of skin damages in Systemic Lupus International Collaborating Clinics/American College of Rheumatology damage index (SLICC/ACR-DI) [7, 8].

Scarring alopecia predominantly affects 20–40-year-old women, with median age 38 years old [9]. Once the scarring established, no treatment is effective to achieve hair regrowth [7]. This irreversible hair loss impacts the appearances of young SLE patients, causing considerable societal costs and reduced quality of life [10]. In addition, it is associated with prolonged disease course [9]. However, to the best of our knowledge, no study has learnt the scarring hair loss in SLE. This case-control study aims to (1) survey the prevalence of scarring alopecia during the disease course of SLE and (2) evaluate the risk factors for scarring alopecia.

## Methods

### Patients

Chinese SLE Treatment and Research group (CSTAR) is the first multi-center Chinese SLE cohort with 104 participating rheumatology centers in 30 provinces in China. We recruited patients who were newly diagnosed SLE in CSTAR and fulfilled the 1997 SLE classification criteria revised by the American Rheumatology Association (ACR) [11] or 2012 SLICC classification criteria for SLE [6]. Patients were excluded if they had no follow-up data or some key baseline data were missing. This study was approved by the Medical Ethics Committee of Peking Union Medical College Hospital, who is the leading site. All patients signed a written informed consent before recruitment.

### Measures

**Fig. 1:**
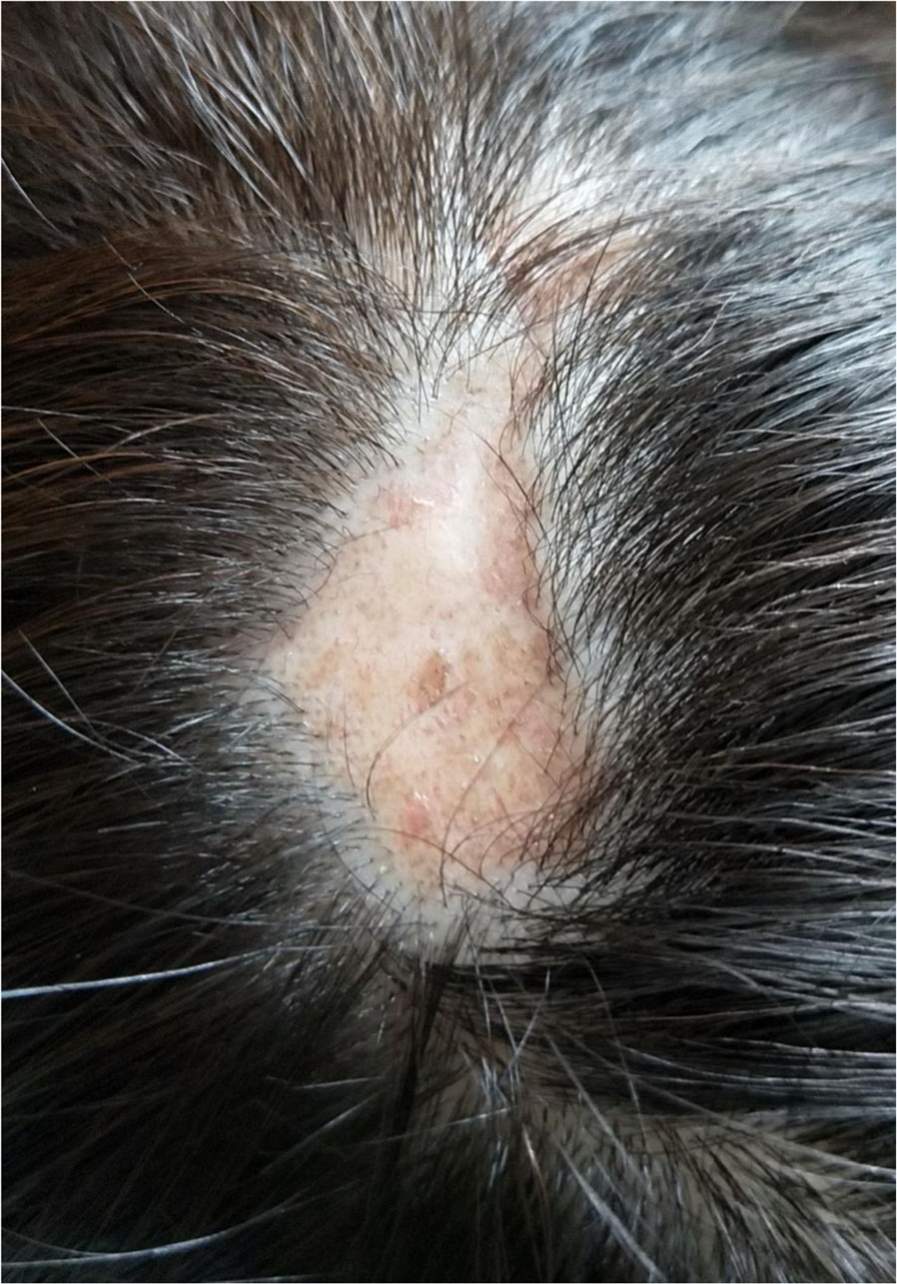
A typical appearance of chronic scarring alopecia from a 30-year-old SLE patient. She suffered from cutaneous lupus erythematosus, alopecia, and leukopenia for 4 years. Finally, her alopecia turned irreversible and scarring

Scarring alopecia was defined according to the SLICC/ACR-DI which was collected during follow-up visit or via self-reported questionnaires. It was a chronic irreversible alopecia presenting for at least 6 months (a typical figure shown in Fig. 1). Non-scarring alopecia was defined in 2012 Systemic Lupus International Collaborating Clinics (SLICC) classification criteria for SLE, which was diffuse thinning or hair fragility with visible broken hairs. We ruled out the patients who had already suffered from scarring alopecia in their registries.

Covariates were collected at registry, including demographic characteristics, autoimmune antibodies, disease activity, and major organ involvements. Demographic characteristics included sex, age, body mass index (kg/m^2^), and comorbidities including smoking (defined as current or suspended in last 5 years), diabetes, hypertension, and dyslipidemia (defined as hypercholesterolaemia ≥ 5.18 mmol/L and/or hypertriglyceridaemia ≥ 1.69 mmol/h).

Autoimmune antibodies were evaluated in antinuclear antibody (ANA), anti-double-stranded DNA (anti-dsDNA), anti-Smith (anti-Sm), anti-Sjogren syndrome A (anti-SSA), anti-Sjogren syndrome B (anti-SSB), anti-ribosomal P protein (anti-rRNP), anti-ribonucleoprotein (anti-RNP), lupus anticoagulant (LA), anticardiolipin antibody (aCL), and anti-β2glycoprotein I (anti-β2GPI).

Disease activity was defined by the systemic lupus erythematosus disease activity index (SLEDAI). Disease damage was assessed by SLICC/ACR-DI (SLICC/ACR-DI excluded chronic scarring alopecia in risk factor analysis).

Major organ involvements included mucocutaneous lesions (acute, subacute, and chronic lupus skin lesions), musculoskeletal involvement (arthritis and myoitis), serositis (pleuritis and pericarditis), renal disorders (24-h urine protein > 0.5 g/24 h or red blood cell casts in urine), neuropsychiatric disorders (as characterized by the ACR nomenclature and case definitions for the 19 neuropsychiatric lupus syndromes [12]), gastrointestinal tract involvement (peritonitis, lupus enteritis, malabsorption, Protein-losing enteropathy, pseudointestinal obstruction, hepatitis, acute cholecystitis, and acute pancreatitis), cardio-respiratory (pulmonary arterial hypertension, myocarditis, arrhythmia, valve dysfunction, pulmonary hemorrhage/pulmonary vasculitis, indirect alveolititis/indirect pneumonia, shrinking lung syndrome, aortitis, and coronaritis), and blood (hemolytic anemia, leukocytopenia, thrombocytopenia, and thrombotic thrombocytopenic purpura).

Treatment strategies were collected when the patients ever used glucocorticoid, immunosuppressants (cyclophosphamide, mycophenolate mofetil, azathioprine, cyclosporine, and tacrolimus), hydroxychloroquine, and biologic agents (TNFi, Rituximab, and Tocilizumab).

### Statistical analysis

Categorical variables were expressed in numbers (%). Continuous variables were presented as mean (SD) or median (IQR). The normality was tested by the Shapiro-Wilk test. We performed univariate analysis on the independent variables to look for statistically significant factors. Factors with *p* values of < 0.10 were included in the multivariate analysis using logistic regression. SPSS (v. 23.0, SPSS Inc., IL, USA) was used for data analysis. Two-sided *p* value less than 0.05 was considered to be statistically significant.

## Results

### Prevalence and baseline characteristics

A total of 4792 patients were recruited. Non-scarring alopecia was observed in 2283 patients (47.64%), while 374 patients (7.80%) suffered scarring alopecia. Among the 374 patients with scarring alopecia, 273 (72.99%) reported non-scarring alopecia at baseline. Table 1 depicted the demographic and clinical characteristics divided by scarring alopecia occurrence. Most patients with scarring alopecia were in their 30s, and 93.89% were female. 4.57% of the patients with scarring alopecia reported smoking, and 10.22% of them had hypertension.

**Table 1:** Demographic and clinical characteristics in patients with or without chronic scarring alopecia

	No scarring alopecia (*n* = 4418)	Scarring alopecia (*n* = 374)	OR (95%CI)	*p* value
Age, years	33.51 (11.18)	33.06 (10.15)	0.996 (0.987~1.006)	0.435
Sex (female)	4148 (93.89%)	344 (91.98%)	0.776 (0.524~1.149)	0.205
Body mass index (kg/m^2^)	21.53 (3.07)	21.37 (2.88)	0.983 (0.946~1.022)	0.382
Comorbidities				
Smoking^a^ (*n* = 4725)	120 (2.76%)	17 (4.57%)	1.689 (1.005~2.839)	0.048*
Diabetes (*n*=4492)	67 (1.62%)	4 (1.10%)	0.678 (0.246~1.869)	0.452
Hypertension(*n*=4699)	506 (11.69%)	38 (10.22%)	0.859 (0.607~1.217)	0.393
Dyslipidemia^b^ (*n* = 4550)	227 (5.43%)	22 (5.98%)	1.108 (0.706~1.740)	0.657
Disease activity and damage				
SLEDAI (mean ± S.D.)	4.67 (5.95)	7.06 (8.26)	–	–
SLICC/ACR-DI (mean ± S.D.)	0.24 (0.61)	0.59 (1.05)	1.241 (1.090~1.414)^c^	0.001**
Autoimmunity characteristics				
ANA positive	4277 (96.81%)	354 (94.65%)	–	–
Anti-dsDNA positive	3229 (73.09%)	277 (74.06%)	1.052 (0.827~1.338)	0.682
Anti-Sm positive	1736 (39.39%)	178 (47.59%)	1.403 (1.135~1.734)	0.002**
Anti-SSA positive (*n* = 3869)	2080 (57.89%)	175 (57.76%)	1.006 (0.793~1.275)	0.964
Anti-SSB positive (*n* = 3856)	740 (20.80%)	67 (22.41%)	0.910 (0.685~1.208)	0.513
Anti-rRNP positive (*n* = 3476)	714 (22.19%)	75 (29.07%)	0.696 (0.525~0.922)	0.011*
Anti-RNP positive (*n* = 3797)	1386 (39.54%)	132 (45.21%)	0.793 (0.624~1.008)	0.058
LA (*n* = 2504)	431 (18.48%)	38 (22.09%)	0.799 (0.549~1.163)	0.242
aCL (*n* = 3320)	545 (17.77%)	45 (17.79%)	0.999 (0.714~1.397)	0.995
Anti-β2GPI (*n* = 3086)	473 (16.53%)	35 (15.56%)	1.075 (0.740~1.563)	0.704
Organ involvements				
Mucocutaneous	2475 (56.02%)	269 (71.93%)	2.011 (1.593~2.540)	< 0.001***
Musculoskeletal	2463 (55.75%)	218 (58.29%)	1.109 (0.896~1.374)	0.342
Serositis	463 (10.48%)	38 (10.16%)	0.966 (0.681~1.370)	0.846
Renal	1518 (34.36%)	98 (26.20%)	0.678 (0.534~0.861)	0.001**
Neuropsychiatric	78 (1.77%)	9 (2.41%)	1.372 (0.683~2.758)	0.375
Gastrointestinal tract	48 (1.09%)	4 (1.07%)	0.984 (0.353~2.745)	0.976
Cardio-respiratory	142 (3.21%)	4 (1.07%)	0.326 (0.120~0.884)	0.028*
Blood	1822 (41.24%)	138 (36.90%)	0.833 (0.670~1.037)	0.101
Treatment strategies (ever use)				
Glucocorticoid	3781 (85.58%)	328 (87.70%)	1.201 (0.872~1.654)	0.261
Immunosuppressants^d^	1831 (41.44%)	111 (29.68%)	0.596 (0.474~0.750)	< 0.001***
Cyclophosphamide	539 (12.20%)	32 (8.56%)	0.673 (0.464~0.978)	0.038*
Mycophenolate mofetil	772 (17.47%)	46 (12.30%)	0.649 (0.472~0.893)	0.008**
Azathioprine	141 (3.19%)	9 (2.41%)	0.698 (0.352~1.383)	0.303
Cyclosporine	252 (5.70%)	17 (4.55%)	0.731 (0.441~1.210)	0.223
Tacrolimus	324 (7.33%)	17 (4.55%)	0.588 (0.357~0.970)	0.038*
Hydroxychloroquine	3449 (78.07%)	298 (79.68%)	1.102 (0.848~1.431)	0.469
Biologic agents	71 (1.61%)	4 (1.07%)	0.662 (0.240~1.822)	0.425

*SLEDAI* SLE disease activity index, *SLICC/ACR-DI* Systemic Lupus International Collaborating Clinics/American College of Rheumatology damage index, *ANA* Antinuclear antibody, *anti-dsDNA* anti-double-stranded DNA, *anti-Sm* anti-Smith, *anti-SSA* anti-Sjogren syndrome A, *anti-SSB* anti-Sjogren syndrome B, *anti-rRNP* anti-ribosomal P protein, *anti-RNP* anti-ribonucleoprotein, *LA* lupus anticoagulant, *aCL* anticardiolipin antibody, *anti-β2GPI* anti-β2glycoprotein I

*p < 0.05; **p < 0.01; ***p < 0.001

^a^Current or suspended in last 5 years

^b^Hypercholesterolaemia ≥ 5.18 mmol/L and/or hypertriglyceridaemia ≥ 1.69 mmol/L

^c^SLICC/ACR-DI excluded chronic scarring alopecia in risk factor analysis

^d^Immunosuppressants included cyclophosphamide, mycophenolate mofetil, azathioprine, cyclosporine, tacrolimus

Regarding disease activity and damage, for patients with scarring alopecia, SLEDAI was 7.06, and SLICC/ACR-DI was 0.59. 47.59% of the patients with scarring alopecia had anti-Sm antibody positive.

In patients with scarring alopecia, the most frequent forms of organ involvements were mucocutaneous (71.93%) and musculoskeletal system (58.29%), followed by hematological disorders (36.90%) and renal involvement (26.20%).

In terms of treatment strategies, 87.70% and 79.68% patients with scarring alopecia ever used glucocorticoid and hydroxychloroquine, respectively. Immunosuppressants were applied in 41.44% and 29.68% of patients with and without scarring alopecia. Among patients with severe organ involvements (renal, neuropsychiatric, gastrointestinal tract, and cardio-respiratory), 1029/1768 (58.20%) received immunosuppressants, while 913/3024 (30.19%) patients without severe organ involvements used these drugs (*p* < 0.001).

### Univariate analysis of risk factors for chronic scarring alopecia

Clinical variables between the two groups, “with scarring alopecia” and “without scarring alopecia”, were compared via univariate analysis. As shown in Table 1, scarring alopecia was significantly associated with smoking (OR 1.689, *p* = 0.048) and SLICC/ACR-DI (OR 1.241, *p* <  0.001). Rates of anti-rRNP (OR 0.696, *p* = 0.002) were lower in patients with scarring alopecia, while rates of anti-Sm (OR 1.403, *p* = 0.002) were higher in alopecia group. The presence of mucocutaneous manifestations (OR 2.011, *p* < 0.001) were positively related to scarring alopecia, renal (OR 0.678, *p* = 0.001) and cardio-respiratory (OR 0.326, *p* = 0.028) system involvements, however, were negatively associated with scarring alopecia. In terms of treatment strategies, immunosuppressants prevented the scarring alopecia occurring.

### Multivariate analysis of risk factors for chronic scarring alopecia

We performed multivariate logistic regression analysis using variables that showed *p* < 0.10 in univariate analysis. As shown in Table 2, high SCLICC/ACR-DI, the presence of anti-Sm and mucocutaneous involvement were risk factors for chronic scarring alopecia in SLE patients. Renal and cardio-respiratory involvements and immunosuppressants showed negatively association with scarring alopecia.

**Table 2:** Multivariate analysis of risk factors associated with scarring alopecia

Risk factors	OR (95%CI)	*p* value
Smoking	1.853 (0.985~3.488)	0.056
SCLICC/ACR-DI (exclude chronic scarring alopecia)	1.409 (1.207~1.646)	< 0.001***
Anti-Sm	1.374 (1.034~1.825)	0.029*
Anti-rRNP	0.808 (0.590~1.108)	0.186
Anti-RNP	1.007 (0.744~1.362)	0.964
Mucocutaneous	2.062 (1.536~2.767)	< 0.001***
Renal	0.714 (0.529~0.964)	0.028*
Cardio-respiratory	0.347 (0.123~0.970)	0.044*
Glucocorticoid	1.196 (0.800~1.789)	0.383
Immunosuppressants^a^	0.675 (0.505~0.901)	0.008**

*SLICC/ACR-DI* Systemic Lupus International Collaborating Clinics/American College of Rheumatology damage index, *ANA* Antinuclear antibody, *anti-Sm* anti-Smith, *anti-rRNP* anti-ribosomal P protein, *anti-RNP* anti-ribonucleoprotein

*p < 0.05; **p < 0.01; ***p < 0.001

^a^Immunosuppressants included cyclophosphamide, mycophenolate mofetil, azathioprine, cyclosporine, tacrolimus

Since SLICC/ACR-DI was revealed to be a positive predictor for scarring alopecia, we further investigated the specific organ damages in patients with scarring alopecia. These patients had relatively high musculoskeletal damage (8.02%) and other skin damages (4.81%), including extensive scarring or panniculum other than scalp and pulp space, and skin ulceration (excluding thrombosis) for > 6 month.

## Discussion

In the present study, we found the prevalence of chronic scarring alopecia in Chinese SLE patients was 7.8%. Mucocutaneous lesions, high SLICC/ACR-DI and positive anti-Sm were risk factors for scarring alopecia, while renal and cardio-respiratory involvements and immunosuppressant treatment were significantly negative associated with it.

Hair loss is a frequent occurrence in SLE, divided as non-scarring and scarring alopecia. Discoid lupus erythematosus (DLE) is the typical clinical manifestation of chronic cutaneous lupus erythematosus, which is the only cutaneous lupus type healing with scarring [13]. In early stage, DLE lesions present inflammatory infiltration. However, patients with scalp DLE proceed towards permanent and irreversible hair loss, once the replacement by fibrous tissue is established [14, 15]. The mechanism for the prevention of hair follicle regeneration and scarring occurring is still unknown. The localization of the hair follicle stem cells to the bulge area of hair follicle is one of explanation for this permanent hair loss process [14]. The inflammatory aggregation in where high concentration of antigen-presenting Langerhans cells associated with hair follicle stem cells leads to the destruction of the sebaceous gland, causing collagenous transformation [16].

DLE happened in 11.2 to 17.6% SLE patients [2], and scarring alopecia has been reported happening in 34 to 35% DLE patients [9, 17]. However, the prevalence of scarring alopecia in SLE is seldom studied. The present study included 4792 SLE patients and demonstrated the prevalence of scarring alopecia 7.80%, which is consistent with a cross-sectional study from Korea [5]. Although the prevalence for scarring alopecia is not that high, given its irreversible situation and reduced life quality of patients, we should learn the risk factors for scarring alopecia and try to prevent it from happening.

Smoking has been found an association with the prevalence or the activity of various autoimmune conditions [18]. Additionally, skin manifestation in SLE has been revealed to be related to tobacco use [19]. Moreover, smoking was found to worsen scalp DLE and make it resistant to treatment [20]. In the present study, we found smoking could be a potential risk factor for scarring alopecia. Smoking cessation should be emphasized for SLE management and scarring alopecia prevention.

Anti-Smith (anti-Sm) antibodies are a highly specific autoantibody in SLE, directly against seven Smith proteins that constitute the small nuclear ribonucleoprotein particles [21, 22]. Besides acting as a SLE classification criterion, anti-Sm antibody has been found to be related with specific subsets of system involvements [23] and disease activity [24]. In terms of skin manifestation, previous research revealed that anti-Sm antibody was associated with photosensitivity, malar rash, and discoid rash [25, 26]. Also, SLE patients with DLE had significantly increased anti-Sm antibody positivity than patients without DLE [27], which is consistent with our study that anti-Sm antibody served as a significant risk factor for scarring alopecia. However, the mechanism behind this association needs further research.

In terms of organ involvements, we found that mucocutaneous lesion was predictors for scarring alopecia, while renal and cardio-respiratory involvements were negatively associated with it. Previous studies indicated that DLE among SLE patients was a marker for less severe disease, with a significantly higher rate for cutaneous manifestations [3] and infrequently occurring in lupus nephritis and other severe organ involvements [27–29]. This was in keeping with our result, which indicated possible different molecular pathways for scarring alopecia and other major organ involvements in SLE patients. In addition, the less frequent severe organ involvement suggested that when effective and strong treatment strategies were adapted to the patients with life-threatening organ involvements, these patients were less likely to develop scarring alopecia.

Immunosuppressive therapies are recommended for severe organ-threatening or life-threatening SLE [30]. Alopecia is a common side effect for immunosuppressive therapy, especially cyclophosphamide [31]. However, the present study revealed that immunosuppressants were negative associated with scarring alopecia. The possible explanations are as followed. First, as mentioned above, patients with major organ involvements had less possibility to present scarring alopecia, so the more usage of immunosuppressive therapies, in some extent, explained this negative association. Second, in spite of the alopecia side effect of these immunosuppressants, these drugs have powerful efficiency in controlling the SLE disease activity and protecting major organ from threatening damage. Additionally, the present study showed that renal and cardio-respiratory involvements, which are indicators for vital systemic involvements, were negatively related to scarring alopecia, indicating the efficacy of immunosuppressive therapy in maintaining low disease activity to prevent scarring alopecia occurring.

Higher SLICC/ACR-DI was demonstrated in our cohort of SLE patients with scarring alopecia. Since we excluded the chronic scarring alopecia factor when calculating SCLICC/ACR-DI in regression analyses, this high score could be attributed to the other skin damages except for scarring alopecia and comorbid musculoskeletal damage. It was demonstrated in previous study that DLE patients had more damage accrual, particularly scarring alopecia, skin scarring, and skin ulcer [28], which explained the co-occurrences of scarring and other skin damages. In terms of musculoskeletal damage, high proportion of DLE patients was reported to have arthritis [29]. However, different results were found in other studies that DLE was associated with reduced risk of arthritis [27, 28] or no association between alopecia and arthritis [3]. In addition, muscle tenderness was revealed to be related with alopecia [3]. To the best of our knowledge, no consensus was made in the association of musculoskeletal damage and scarring alopecia. In the present study, our results suggested that skin and musculoskeletal damages in SLE patients may share the same molecular pathways, which needs further investigation.

Some limitations of the present study should be addressed. First, this is a preliminary and exploratory case control study, lacking the record of the exact scarring alopecia occurrence time point, and the self-report questionnaires could cause bias to the ascertainment of scarring alopecia. Second, to our knowledge, this is the only study to present the prevalence and risk factors for scarring alopecia in SLE patients and was conducted only in Chinese SLE patients. The results should be verified in other SLE cohort population.

## Conclusions

In summary, the prevalence of scarring alopecia in SLE patients is 7.80%. Positive anti-Sm antibody, mucocutaneous involvement, and high SLICC/ACR-DI were risk factors for scarring alopecia, while renal and cardio-respiratory involvements and immunosuppressant usage were significantly negatively associated. Active treatment strategies should be adopted to prevent scarring alopecia in SLE patients.

## Data Availability

All data generated or analyzed during this study are included in this article.

## Abbreviations

SLE: Systemic lupus erythematosus
CSTAR: Chinese SLE Treatment and Research Group
DLE: Discoid lupus erythematosus
SLEDAI: SLE disease activity index
SLICC/ACR-DI: Systemic Lupus International Collaborating Clinics/American College of Rheumatology damage index
ANA: Antinuclear antibody
anti-dsDNA: Anti-double-stranded DNA
anti-Sm: Anti-Smith
anti-SSA: Anti-Sjogren syndrome A
anti-SSB: Anti-Sjogren syndrome B
anti-rRNP: Anti-ribosomal P protein
anti-RNP: Anti-ribonucleoprotein
LA: Lupus anticoagulant
aCL: Anticardiolipin antibody
anti-β2GPI: Anti-β2glycoprotein I
